# Gluten Detection and Speciation by Liquid Chromatography Mass Spectrometry (LC-MS/MS)

**DOI:** 10.3390/foods3010013

**Published:** 2013-12-23

**Authors:** Stephen Lock

**Affiliations:** AB SCIEX, Pheonix House, Centre Park, Warrington, WA1 1RX, UK; E-Mail: Stephen.lock@absciex.com; Tel.: +44-772-027-6948

**Keywords:** gluten, speciation, LC-MS/MS

## Abstract

Liquid chromatography tandem mass spectrometry (LC-MS/MS) has been used historically in proteomics research for over 20 years. However, until recently LC-MS/MS has only been routinely used in food testing for small molecule contaminant detection, for example pesticide and veterinary residue detection, and not as a replacement of microbiological food testing methods, specifically allergen analysis. Over the last couple of years, articles have started to be published which describe the detection of allergens by LC-MS/MS. In this article we will describe how LC-MS/MS can be applied in the area of gluten detection and how it can be used to specifically differentiate the species of gluten used in food, where specific markers for each variety of gluten can be simultaneously acquired and detected at the same time. The article will discuss the effect of variety on the peptide response observed from different wheat grain varieties and will describe the sample preparation protocol which is essential for generating the peptide markers used for speciation.

## 1. Introduction

Gluten is known to produce an allergic response, and intolerance to gluten leads to celiac disease. Levels of intolerance to gluten often vary with gluten variety; this is especially relevant to the use of oats which has a low effect on celiac suffers. Most current ELISA methodology, with the exception of assays based on the R5 antibody [1], detect the presence of barley, rye and wheat but also oats. Assays based on the R5 antibody are not sensitive to oats, but this assay still cannot differentiate barley, wheat and rye. Also, all the ELISA methods that detect gluten are based on one section of the gluten protein (for example, in the case of the R5 assay, the peptide sequence glutamine-glutamine-proline-phenylalanine-proline (QQPFP) peptide epitope) and as such are susceptible to false positives and false negatives. Liquid chromatography tandem mass spectrometry (LC-MS/MS) has the ability to detect species based on multiple markers with multiple points of confirmation which makes it far less susceptible to producing false negatives and positives, and gives far more confirmation in detection. Due to its very specific nature, it is also capable of distinguishing species by using multiple peptide markers as shown previously [2,3,4,5]. A suitable LC-MS/MS method that could offer the possibility to differentiate between gluten species with a high degree of specificity would be beneficial to both grain producers and consumers.

In addition, legislation is changing with respect to gluten and, in the UK, one of the first allergen limits came into effect on 1 January 2012 [6,7]. On this date the laws governing food labeling were changed such that three different terms can now be used:

1. Gluten-free—is covered by the law and applies only to food which has 20 parts per million (ppm) or less of gluten.
2. Very low gluten—is covered by the law and is for foods which have between 21 and 100 ppm.
3. No gluten-containing ingredients—this is not covered by the law and is for foods that are made with ingredients that do not contain gluten and where cross contamination controls are in place. These foods will have very low levels of gluten, but have not been tested to the same extent as those labeled gluten-free or very low gluten.

These changes in the food labeling law have recently been followed in the US by the FDA [8]. Some initial studies using LC-MS/MS in gluten profiling were presented in 2012 [9]. In these studies trypsin was used as the enzyme for digestion, even though gluten proteins do not undergo a large level of trypsin digestion (due to a low number of lysine and arginine residues) some unique marker peptides were found. Recently, chymotrypsin has also been used as an alternative to trypsin [10] to generate a large number of peptides for the improved detection of wheat gliadin proteins, but this method used longer digestion times and labelling chemistry to better characterise the gluten proteins. 

The purpose of this study was a follow up to the original poster presentation in 2012 [9] to investigate a simpler approach to preparing extracts and use recent advances in LC technology to help reach detection limits below the requirements of the current labelling legislation. One of the main purposes of this work was to develop an approach which could analyse a sample in one day using inexpensive available chemicals. In this study single varieties of grain which have not been first milled were ground into flour using a commercial coffee grinder. These samples, together with commercial samples of self-raising flour, gluten-free flour and some gluten and gluten-free foods, were extracted and then the allergenic proteins were reduced, alkylated and digested using trypsin. In this study the extracts produced were simply diluted into 0.1 % formic acid prior to injection and separation by reverse phase chromatography and LC-MS/MS detection. The LC used was a Eksigent ekspert™ microLC 200 UHPLC system (Eksigent, Redwood City, CA, USA) which had been previously evaluated for the detection of egg and milk allergens in wine [11] and had been shown to offer a 5-fold improvement in sensitivity. The mass spectrometry methods utilised *Scheduled* MRM™ (an algorithm which allows the independent monitoring of MRM transitions with a defined window around the expected retentions time for each MRM transition which is available in the Analyst^®^ software version 1.5 and onwards from AB SCIEX) for multiple peptides for each gluten species, so that presence of allergen can be unambiguously confirmed. 

## 2. Experimental Section

The method described is based on the classic proteomics sequencing approach which involves first the extraction of the protein from a matrix. Once extracted, the proteins are reduced, alkylated and digested. The extracts were finally diluted and analyzed by LC-MS/MS using an AB SCIEX QTRAP^®^ 4500 LC/MS/MS system (AB SCIEX, Warrington, UK).

### 2.1. Preparation of Tryptic Digests

#### 2.1.1. Extraction of Proteins

Markers proteins from wheat, oats, barley and rye were extracted by placing powdered sample (0.5 g of flour or cookie which had been ground using a commercial coffee grinder) into a falcon tube (15 mL) with extraction buffer [5 mL of a 50:50 mixture of ethanol containing 2 M urea and 50 mM 2-amino-2-hydroxymethyl-propane-1,3-diol (Tris)]. This mixture was shaken by hand (30 s) and then heated and shaken in an orbital water bath (40 °C, 60 min).

#### 2.1.2. Reduction and Alkylation of Proteins

Once extracted the samples were centrifuged (2500 rpm, 5 min, 20 °C). The supernatant (0.5 mL) was then reduced by the addition of TCEP [tris(2-carboxyethyl)phosphine, 0.2 M, 50 µL, 60 °C, 60 min in a thermal mixer] and cooled to room temperature. MMTS (methyl methanethiosulfonate, 0.2 M, 100 µL) was added and the sample left in the dark (30 min) to alkylate the free cysteine residues.

#### 2.1.3. Tryptic Digestion of Proteins

Once the proteins had been alkylated the sample were diluted with buffer (1.35 mL, 0.1 M ammonium bicarbonate solution) and trypsin (80 µL, 0.5 mg/mL, Sigma Aldrich part number 93614) was added. The proteins were then digested for one hour (Eppendorf thermal mixer model number 21516-170, 40 °C, Eppendorf, Stevenage, UK). The digestion was quenched by taking the digest extract (100 µL) and adding 0.1% formic acid (300 µL). The sample was centrifuged (13,000 rpm, 5 min) and then the supernatant was injected into the LC-MS/MS system.

### 2.2. LC-MS/MS Analysis of Tryptic Digests

All analyses was done using an Eksigent ekspert™ microLC 200 UHPLC system (Eksigent, Redwood City, CA, USA). The extracts (10 µL injection, full loop fill mode) were separated on a reversed-phase Triart C18 column (100 × 0.5 mm, 2.7 μm, YMC, Dinslaken, Germany) at a temperature of 40 °C using the gradient conditions shown in Table 1 where A was water, B was acetonitrile with both phases containing 0.1% formic acid. Micro LC was used as it had previously been shown to improve responses in peptide analysis using electrospray ionization by over 5 fold [11].

**Table 1:** Gradient elution used for analysis of extracts.

Step	Time (mins)	Flow rate	% A	% B
1	1	25 μL/min	95	5
2	6	25 μL/min	75	25
3	8	25 μL/min	5	95
4	9	25 μL/min	5	95
5	9.2	25 μL/min	95	5
6	12	25 μL/min	96	5

**Figure 1:**
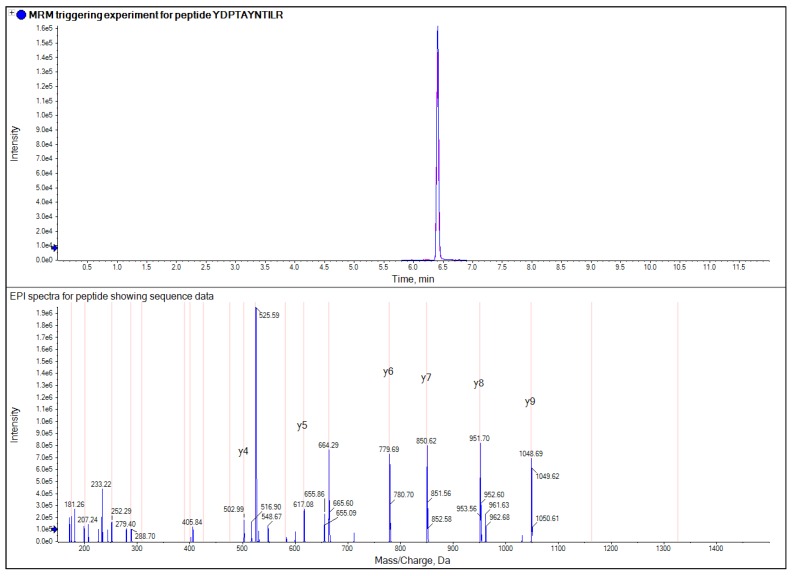
Example of a MIDAS experiment where the top pane shows a MRM trace for a wheat peptide from a flour extract and the bottom pane shows the triggered enhanced product ion (EPI) spectra for the peptide which contains sequence data confirming its identity.

All analyses were performed on an AB SCIEX QTRAP^®^ 4500 LC/MS/MS system (AB SCIEX, Warrington, UK) using electrospray ionization (ESI). The initial method development was carried out using the MIDAS™ workflow (MRM-initiated detection and sequencing [12]) and for microLC analysis the electrode was changed to a microLC hybrid electrode (25 μm ID) designed for microLC [13]. For MIDAS a set of predicted MRM transitions from the known protein sequence were used as a survey scan to trigger the acquisition of EPI spectra (acquired at a scan speed of 10,000 amu/s with dynamic fill time and rolling collision energy active and Q1 resolution set to low) an example of this is shown in Figure 1. 

This MIDAS data was submitted to a database search engine for confirmation of peptide identification and to test the feasibility of the MRM transitions for gluten and species identification. With this workflow MRM transitions were designed without the need for synthetic peptides. In the final micro LC method the Turbo V™ source conditions used were gas 1, gas 2 and the Curtain Gas™ interface set to 30 psi, the temperature of the source was set at 350 °C and the IS voltage was 5500 V. The peptides were analyzed using the *Scheduled* MRM™ algorithm with an MRM detection window of 60 s and a target scan time of 0.30 s. Q1 resolution was set to low and Q3 resolution was set to unit. MRM transitions shown in Table 2 were evaluated for rye, oats, wheat and barley, and each MRM transition used the same declustering voltage (80 V) and entrance potential (10 V). These MRM transitions corresponded to the peptides shown in Table 3.

**Table 2:** MRM transitions used for triggers for generating EPI spectra and peptide detection.

Peptide ID	Q1 mass (amu)	Q3 Mass 1 (amu)	Q3 Mass 2 (amu)	Q3 Mass 3 (amu)	RT (mins)	CE (V)	CXP (V)
Wheat 1	557.3	886.5	548.7	787.4	4.2	32	12
Wheat 2	579.4	897.6	711.5		7.5	33	12
Wheat 3	458.8	730.4	560.3	458.8	3.9	28	12
Wheat 4	594.8	792.4	978.5	538.9	7.1	34	12
Wheat 5	663.8	850.5	779.4	951.7	6.4	37	12
Wheat 6	538.3	547.3	776.4	705.4	4.1	34	12
Barley 1	835.4	947.5	1096.5	1227.6	7.4	47	10
Barley 2	336.2	515.3	554.3	497.3	5.5	13	10
Barley 3	820.4	1096.5	548.3	713.4	7.4	43	10
Barley 4	499.3	785.5	575.3	393.3	6.3	24	10
Barley 5	855.6	980.5	642.4		7.3	38	10
Oats 1	989	997.6	1084.7	1233.7	7.5	54	10
Oats 2	777.4	984.4	1112.5	1225.6	6.1	38	10
Oats 3	627.3	642.4	1012.5		5.8	33	10
Oats 4	365.1	601.2	473.1		3.8	23	10
Rye 1	937	1177.6			7.5	46	10
Rye 1	625	941.6			7.5	24	10
Rye 2	851.7	1199.7	1071.6	1210.2	7.3	43	10
Rye 3	997	1225.6			7.4	46	10
Rye 3	665	1225.6	1128.5		7.4	29	10
Rye 4	988	1197.7	1100.6		7.5	45	10
Rye 4	659	1197.7			7.5	29	10

**Table 3:** Marker peptides and sequence information used for gluten species markers (the peptide information was taken from searches of the Swiss-Prot database [14]).

Species	Peptide			Protein			Entry number			Peptide sequence
*Hordeum* *vulgare* (barley)	1			B1-hordein			P06470			TLPMMCSVNVPLYR
	2			B1-hordein			P06470			GVGPSVGV
	3			B3-hordein			P06471			TLPTMCSVNVPLYR
	4			B3-hordein			P06471			IVPLAIDTR
	5			B3-hordein			P06471			SQMLQQSSCHVLQQ QCCQQLPQIPEQLR
*Avena sativa* (oats)	1			Avenin-3			P80356			QFLVQQCSPVAVVPFLR	
	2			Avenin-3			P80356			SQILQQSSCQVMR
	3			Avenin-3			P80356			QLEQIPEQLR
	4			Avenin-3			P80356			QQCCR
*Secale cereale* (rye)	1			75k gamma secalin			E5KZQ3			NVLLQQCSPVALVSSLR	
	2			75k gamma secalin			E5KZQ4			EGVQILLPQSHQQHVGQGAL AQVQGIIQPQQLSQLEVVR	
	3			75k gamma secalin			E5KZQ5			SLVLQNLPTMCNVYVPR	
	4			75k gamma secalin			E5KZQ5			QCSTIQAPFASIVTGIVGH	
*Triticum aestivum* (wheat)	1			Glutenin, subunit DY10			P10387			QVVDQQLAGR	
	2			Glutenin, subunit PW212			P08489			IFWGIPALLK	
	3			Glutenin, subunit DY10			P10387			SVAVSQVAR	
	4			Glutenin, subunit DY10			P10387			LPWSTGLQMR	
	5			Beta-amylase			P93594			YDPTAYNTILR	
	6			Alpha-amylase inhibitor 0.19			P01085			EHGAQEGQAGTGAFPR	

## 3. Results

**Figure 2:**
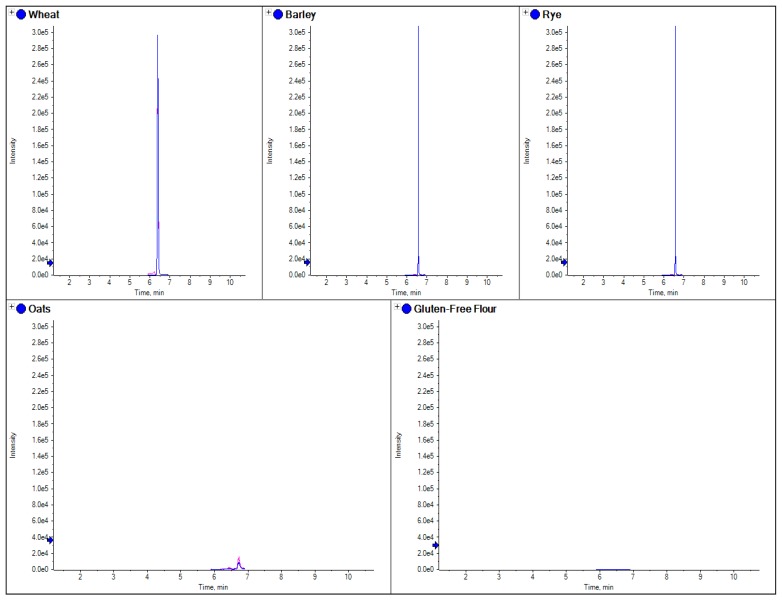
The comparison of separate extracts from barley, wheat, rye, oats and gluten free flour. Here the chromatograms for three MRM transitions for a specific marker peptide from beta amylase (wheat 5 in Table 3) have been shown for each species.

To test this approach, several samples of grain from single varieties of wheat, rye, barley and oats, together with commercial samples of gluten-free flour, oats cookies, gluten free cookies, wheat cookies and a self-raising flour (from a local supermarket in the UK) were collected. Each sample of grain was milled in a commercially available coffee bean grinder to make single variety flour, and all these samples were extracted and analyzed using the described method. Figure 2 shows the comparisons of the four different grain flours using a beta amylase marker peptide. 

**Figure 3:**
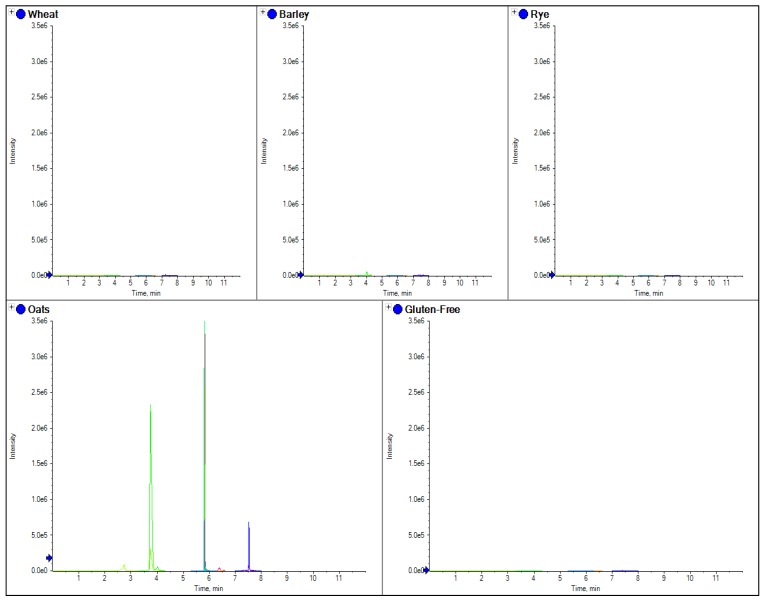
The comparison of separate extracts from barley, wheat, rye, oats and gluten free flour. Here the overlaid chromatograms for oats marker peptides (obtained from the theoretical digestion of avenin) have been shown for flour extract for each sample.

**Figure 4:**
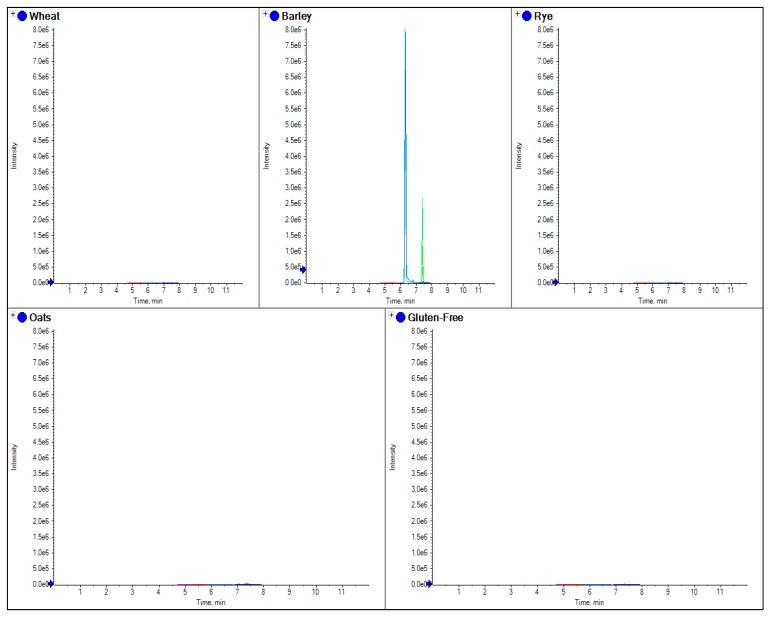
The comparison of separate extracts from barley, wheat, rye and oats flour. Here the overlaid chromatograms for barley marker peptides (obtained from the theoretical digestion of hordein) have been shown for flour extracts for each sample.

**Figure 5:**
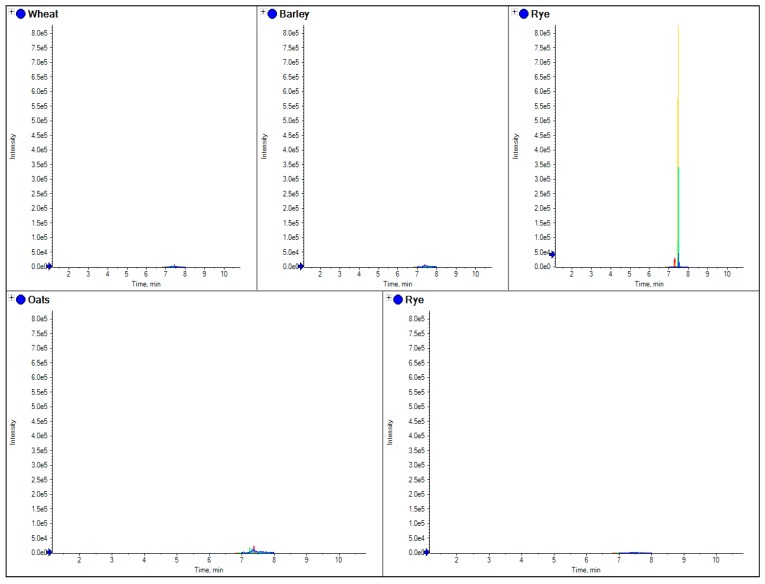
The comparison of separate extracts from barley, wheat, rye and oats flour. Here the overlaid chromatograms for rye marker peptides (obtained from the theoretical digestion of secalin) have been shown for flour extracts for each sample.

**Figure 6:**
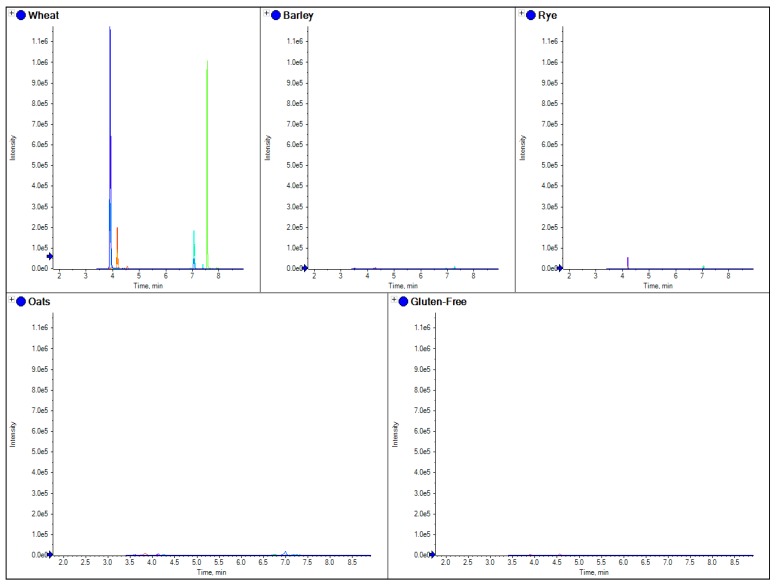
The comparison of separate extracts from barley, wheat, rye, oats and gluten-free flour. Here four separate peptide chromatograms for wheat marker peptides (obtained from the theoretical digestion of glutenin) have been shown for the extracts of each sample.

Separate marker peptides were also tested for oats, barley, wheat and rye, which were specific for each species; these are shown in Figure 3, Figure 4, Figure 5 and Figure 6.

**Figure 7:**
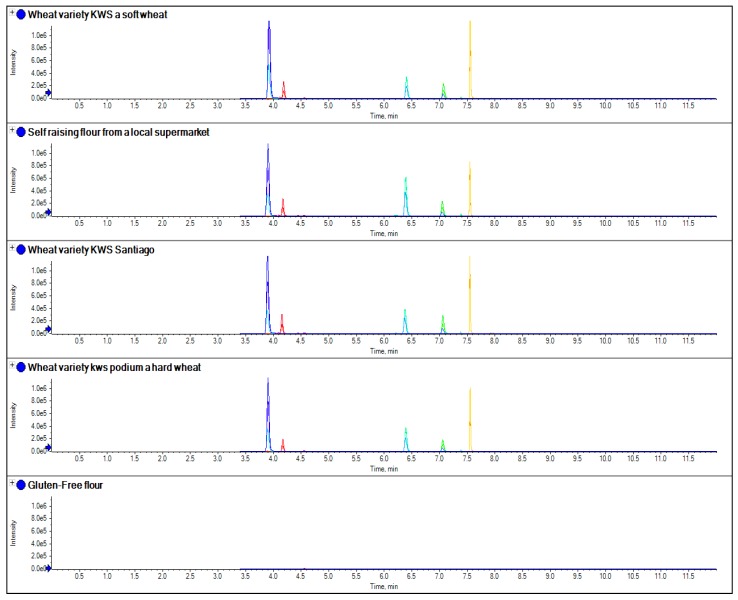
The comparison of separate extracts of several samples of wheat obtained from single variety grain samples, as well as a sample of gluten-free flour and self-raising flour obtained from a local supermarket using the wheat peptides in Table 3.

**Figure 8:**
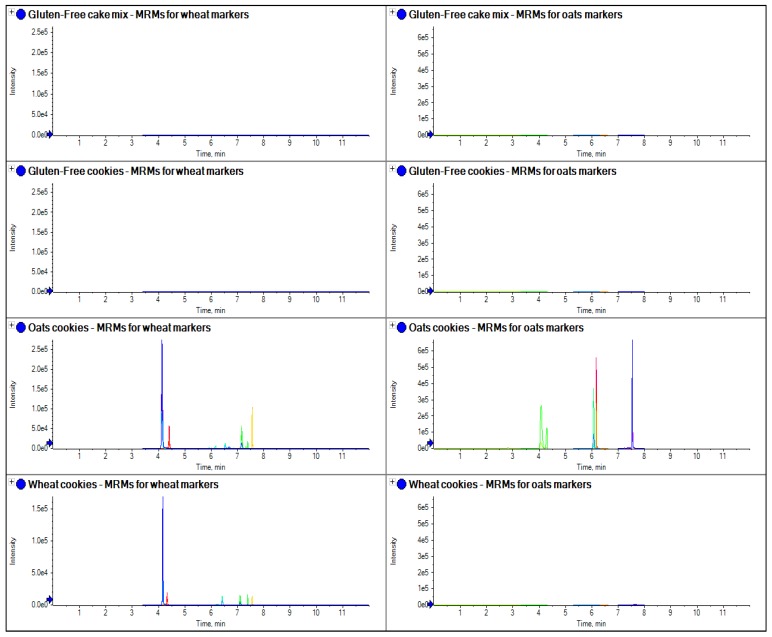
The comparison of extracts from several samples of food collected from a local supermarket and analyzed for gluten markers for oats and wheat.

**Figure 9:**
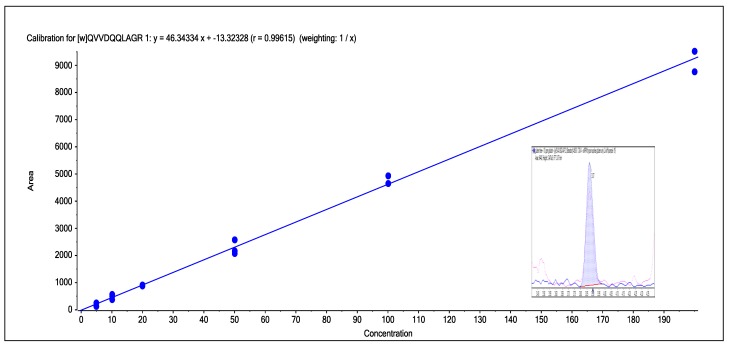
The calibration line obtained from the spiking of gliadin into gluten-free wheat from the range of 5–200 ppm for wheat peptide 3. Inlayed in the calibration line is the chromatogram for the 10 ppm spike of gliadin into gluten-free flour. The calibration line was a linear 1/*x* fit with *r* = 0.9944.

To further evaluate this approach, three samples of single varieties of wheat grain were obtained and extracted and compared with an extraction of gluten-free flour (a mix of tapioca, buckwheat, rice, maize and potato flour), as well as a sample of self-raising flour obtained from a local supermarket (Figure 7). The method was further evaluated by applying the same extraction and analysis method to a sample of gluten-free cake mix and cookies, as well as samples of oats and wheat cookies, to see if it could be applied to processed food (Figure 8). To assess linearity and sensitivity, samples of gluten-free flour were spiked at different levels with gliadin protein from wheat, which had been purchased from Sigma Aldrich (part number G3375, Figure 9).

## 4. Discussion

Similar to current R5 antibody based ELISA methods, LC-MS/MS marker peptides can be found which are present in all gluten varieties, with the exception of oats as shown in Figure 2, where the use of a tryptic peptide from the protein beta amylase was present in wheat, barley and rye, but absent in oats. This marker gave a consistent MRM ratio for the three peptide MRM transitions across all three species and is a good marker to replicate the results of R5 antibody ELISA based methods which are positive to only wheat, rye and barley. However, LC-MS/MS differs to ELISA in that specific markers from the individual species of oats, rye, barley and wheat can also be developed. Figure 3, Figure 4, Figure 5 and Figure 6 show how individual markers for each of these species can be used to distinguish different species by LC-MS/MS and specifically confirm whether oats had been used as a replacement to wheat. LC-MS/MS offers an advantage over ELISA based methods in that you can use multiple peptide markers with multiple MRMs for each peptide to confirm the presence of the gluten species in the sample. One question that had been asked was that if LC-MS/MS was so specific, would it be affected by the variety of the species in the sample? To test this hypothesis, several single varieties of wheat grain were obtained from a grain supplier. These were then ground and the resulting flour extracted to test the effect of variety of wheat on the peptides detected. In Figure 7 the comparison of four peptide markers for wheat, across different samples of flour, were compared. What is immediately apparent is that all the peptides are seen in all the samples with the exception of the gluten-free flour. These wheat varieties included commercial self-raising flour obtained from a supermarket, as well as hard and soft wheat varieties. From the peptide responses, you can also see that the relative responses are the same for three out of the four, with only one marker significantly higher in variety 3, but this may have been as a result of the change in matrix interference for this particular sample. This clearly indicates that the majority of LC-MS/MS markers which have been found are independent of variety used to produce the wheat flour and are just species specific.

One of the important tests for the feasibility of the use of LC-MS/MS was its ability to detect gluten in processed food. In processed food, ELISA kits have been shown to fail to pick up allergens due to processing changes in the protein structure which then prevent the antibody binding and this leads to false negatives [15]. Also, due to the fact that ELISA methodology just relies on one protein region, unspecific binding has also been shown which has led to false positives in some instances [16]. In this work, the LC-MS/MS method was applied to some cookies as well as gluten-free flour to determine its ability to detect the markers in processed food. In Figure 8 it can clearly be seen that LC-MS/MS can detect the markers in the processed food and distinguish between varieties. In the case of the oats cookie, wheat and oats had been used in its manufacture and markers for both varieties were detected (barley and rye were not present in any sample, although not shown in this figure). However, in the wheat cookie, only wheat was used and this is the only species detected. In the gluten-free products, no gluten markers were detected.

A final test was linearity of response and the sensitivity of the method. To test both of these, gluten-free flour was spiked with gliadin (wheat protein obtained commercially) from a range of 5–200 ppm. A calibration line, shown in Figure 9, for one of the peptides clearly shows that the LC-MS/MS response obtained for this wheat marker was linear—this was typical of the other markers used for wheat—and for this marker a 10 ppm spike could be easily detected. Marker peptides therefore could be detected at 5–10 ppm levels in the spiked sample of gluten-free flour even though the current sample preparation used an 80-fold dilution of the original sample.

## 5. Conclusions

This work has demonstrated that LC-MS/MS can be used to detect gluten in processed food and food ingredients. The work demonstrated that markers can be obtained which are specific for each individual species of gluten. The presence of these multiple markers for individual species were not variety dependent, as shown in a test of several single varieties of wheat flour (where all the same markers were detected), but some were species-dependent. As well as species dependent markers, markers for proteins that are present in rye, wheat and barley, but absent in oats, can also be added to the method to mimic the behavior of the R5 antibody based ELISA method to generally pick up the species that are high in gluten and affect people who suffer from Celiac disease. The method has been shown to detect levels of 5–10 ppm gluten proteins in gluten-free flour and offers an extended linear response which is envisaged to be a lot larger than that normally obtained for ELISA assays. Further to this, as the current method actually involves an 80-fold dilution of the sample, before injecting onto the LC-MSMS system, it offers the potential of detecting low ppm (0.5–5 ppm) when an SPE protocol is used to collect concentrate and purify the peptide markers.

The presence of multiple markers for each gluten variety and the potential of acquiring MRM triggered product ion scans [12], offers multiple points for confirmation of gluten contamination and provide confidence in the results, and reduces the risk of false positives and false negatives which can occur in ELISA assays.
